# Diaqua­bis­(1*H*-imidazole-4-carboxyl­ato-κ^2^ *N* ^3^,*O* ^4^)manganese(II)

**DOI:** 10.1107/S1600536813004091

**Published:** 2013-02-28

**Authors:** Zhi-Yong Xiong, Lin Li, Xiang-Jie Zhao, Hai-Ming Chen

**Affiliations:** a College of Light Industry and Food Sciences, South China University of Technology, Guangzhou 510641, People’s Republic of China; b Engineering Research Center of Starch and Vegetable Protein Processing, Ministry of Education, South China University of Technology, Guangzhou 510640, People’s Republic of China; c School of Chemical Engineering and Materials Science, Beijing Institute of Technology, Zhuhai 519088, People’s Republic of China

## Abstract

In the title compound, [Mn(C_4_H_3_N_2_O_2_)_2_(H_2_O)_2_], the Mn^II^ ion is located on a twofold rotation axis and displays a distorted octa­hedral coordination environment, defined by two *N*,*O*-bidentate 1*H*-imidazole-4-carboxyl­ate ligands in the equatorial plane and two water mol­ecules in axial positions. In the crystal, O—H⋯O and N—H⋯O hydrogen bonds link the mol­ecules into a three-dimensional supra­molecular network. π–π stacking inter­actions between the imidazole rings [centroid–centroid distances = 3.5188 (15) and 3.6687 (15) Å] further stabilize the structure.

## Related literature

For related structures, see: Cai *et al.* (2012 ▶); Chen (2012 ▶); Gryz *et al.* (2007 ▶); Haggag (2005 ▶); Shuai *et al.* (2011 ▶); Starosta & Leciejewicz (2006 ▶); Yin *et al.* (2009 ▶); Zheng *et al.* (2011 ▶).
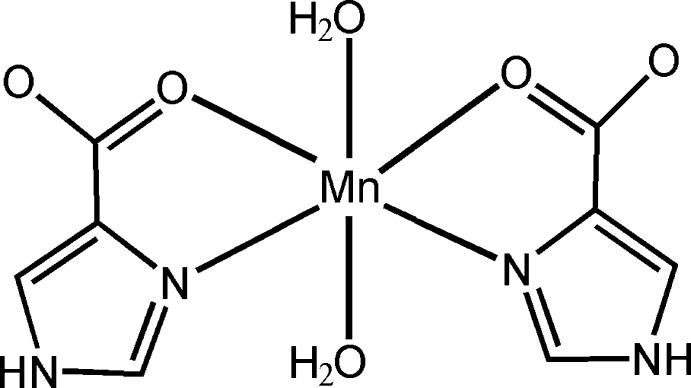


## Experimental

### 

#### Crystal data

[Mn(C_4_H_3_N_2_O_2_)_2_(H_2_O)_2_]
*M* *_r_* = 313.14
Orthorhombic, 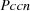

*a* = 7.3052 (10) Å
*b* = 11.7997 (17) Å
*c* = 13.5156 (19) Å
*V* = 1165.0 (3) Å^3^
*Z* = 4
Mo *K*α radiation
μ = 1.16 mm^−1^
*T* = 298 K
0.36 × 0.32 × 0.30 mm

#### Data collection

Bruker APEXII CCD diffractometer
Absorption correction: multi-scan (*SADABS*; Bruker, 2001 ▶) *T*_min_ = 0.679, *T*_max_ = 0.721
5775 measured reflections
1145 independent reflections
972 reflections with *I* > 2σ(*I*)
*R*_int_ = 0.067

#### Refinement

*R*[*F* ^2^ > 2σ(*F* ^2^)] = 0.039
*wR*(*F* ^2^) = 0.109
*S* = 1.07
1145 reflections
87 parameters
H-atom parameters constrained
Δρ_max_ = 0.34 e Å^−3^
Δρ_min_ = −0.57 e Å^−3^

### 

Data collection: *APEX2* (Bruker, 2007 ▶); cell refinement: *SAINT* (Bruker, 2007 ▶); data reduction: *SAINT*; program(s) used to solve structure: *SHELXS97* (Sheldrick, 2008 ▶); program(s) used to refine structure: *SHELXL97* (Sheldrick, 2008 ▶); molecular graphics: *ORTEPIII* (Burnett & Johnson, 1996 ▶) and *PLATON* (Spek, 2009 ▶); software used to prepare material for publication: *SHELXL97*.

## Supplementary Material

Crystal structure: contains datablock(s) I, global. DOI: 10.1107/S1600536813004091/hy2616sup1.cif

Structure factors: contains datablock(s) I. DOI: 10.1107/S1600536813004091/hy2616Isup2.hkl

Additional supplementary materials:  crystallographic information; 3D view; checkCIF report

## Figures and Tables

**Table 1:** Hydrogen-bond geometry (Å, °)

*D*—H⋯*A*	*D*—H	H⋯*A*	*D*⋯*A*	*D*—H⋯*A*
N2—H2⋯O2^i^	0.86	1.95	2.811 (3)	173
O1*W*—H1*WA*⋯O2^ii^	0.87	1.96	2.818 (2)	167
O1*W*—H1*WB*⋯O2^iii^	0.73	2.02	2.751 (2)	176

Symmetry codes: (i) 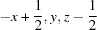; (ii) 
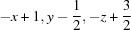
; (iii) 
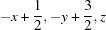
.

**Fig. 1.**
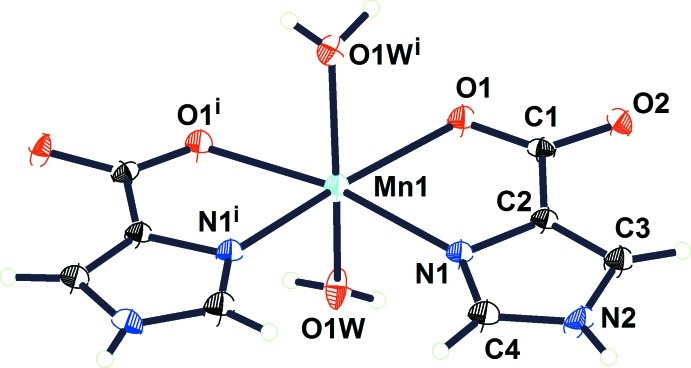
The molecular structure of the title compound. Displacement ellipsoids are drawn at the 30% probability level. [Symmetry code: (i) -x+3/2, -y+3/2, z.]

**Fig. 2.**
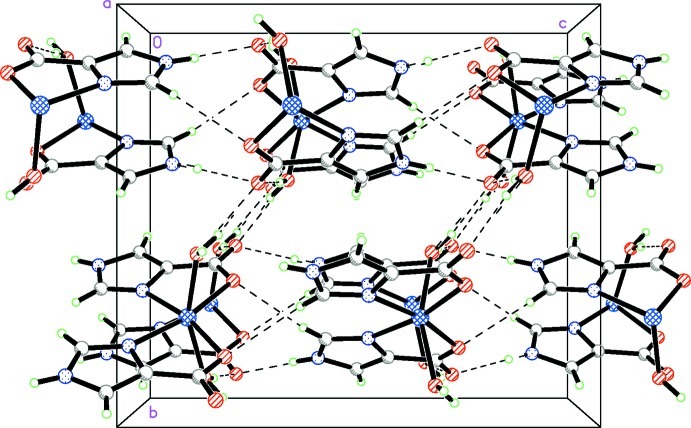
The crystal packing of the title compound, showing the three-dimensional supramolecular network. Hydrogen bonds are shown as dashed lines.

## supplementary crystallographic information

### Comment

In the past few years, structures containing metals and *N*-heterocyclic carboxylic acids have attracted much attention due to their fascinating structures and potential applications in many fields. 1*H*-Imidazole-4-carboxylic acid (H_2_imc), which contains two N atoms of an imidazole group and one carboxylate group, has been widely used to prepare a variety of coordination polymers with different structures and exceptional properties (Cai *et al.*, 2012; Gryz *et al.*, 2007; Haggag, 2005; Starosta & Leciejewicz, 2006; Zheng *et al.*, 2011). For instance, three mononuclear complexes, [Cd(Himc)_2_(H_2_O)_2_] (Yin *et al.*, 2009), [Zn(Himc)_2_(H_2_O)_2_] (Shuai *et al.*, 2011) and [Co(Himc)_2_(H_2_O)_2_] (Chen, 2012), have been reported. In this paper, we report the synthesis and structure of a new Mn(II) coordination polymer, [Mn(Himc)_2_(H_2_O)_2_], which is isomorphous with the Cd(II), Zn(II) and Co(II) analogs.

The asymmetric unit of the title compound contains a half of [Mn(Himc)_2_(H_2_O)_2_] formula unit. The Mn^II^ ion, lying on a twofold rotation axis, is six-coordinated by two N and two O atoms from two *cis*-oriented *N,O*-bidentate Himc ligands in the equatorial plane, and two water molecules in the axial positions, forming a slightly distorted octahedral geometry (Fig. 1). The bond lengths and angles around the Mn atom are normal. In the crystal structure, intermolecular O—H···O and N—H···O hydrogen bonds (Table 1) involving the coordinated water O atoms, carboxylate O atoms and imidazole N atoms link the molecules into a three-dimensional supramolecular network, as presented in Fig. 2. π–π stacking interactions between the imidazole rings [centroid–centroid distances = 3.5188 (15) and 3.6687 (15) Å] further stabilize the crystal structure.

### Experimental

A mixture of H_2_imc (0.30 mmol), MnCl_2_.6H_2_O (0.30 mmol) and 6 ml EtOH/H_2_O (v/v 1:1) was sealed into a 10 ml sample bottle reactor and heated at 373 k for 72 h under autogenous pressure, and then slowly cooled to room temperature at a rate of 5 K/h. Colorless block crystals of the title compound were obtained, washed with distilled water and dried in air (yield: 30%).

### Refinement

C- and N-bound H atoms were positioned geometrically and refined using a riding model, with C—H = 0.93 and N—H = 0.86 Å and with *U*_iso_(H) = 1.2*U*_eq_(C, N). H atoms of the water molecule were located from a difference Fourier map and refined as riding atoms, with *U*_iso_(H) = 1.5*U*_eq_(O).

### Crystal data

**Table:**

[Mn(C_4_H_3_N_2_O_2_)_2_(H_2_O)_2_]	*F*(000) = 636
*M**_r_* = 313.14	*D*_x_ = 1.785 Mg m^−^^3^
Orthorhombic, *P**c**c**n*	Mo *K*α radiation, λ = 0.71073 Å
Hall symbol: -P 2ab 2ac	Cell parameters from 1516 reflections
*a* = 7.3052 (10) Å	θ = 3.3–24.9°
*b* = 11.7997 (17) Å	µ = 1.16 mm^−^^1^
*c* = 13.5156 (19) Å	*T* = 298 K
*V* = 1165.0 (3) Å^3^	Block, colourless
*Z* = 4	0.36 × 0.32 × 0.30 mm

### Data collection

**Table:**

Bruker APEXII CCD diffractometer	1145 independent reflections
Radiation source: fine-focus sealed tube	972 reflections with *I* > 2σ(*I*)
Graphite monochromator	*R*_int_ = 0.067
φ and ω scans	θ_max_ = 26.0°, θ_min_ = 3.0°
Absorption correction: multi-scan (*SADABS*; Bruker, 2001)	*h* = −8→9
*T*_min_ = 0.679, *T*_max_ = 0.721	*k* = −14→10
5775 measured reflections	*l* = −16→15

### Refinement

**Table:**

Refinement on *F*^2^	Primary atom site location: structure-invariant direct methods
Least-squares matrix: full	Secondary atom site location: difference Fourier map
*R*[*F*^2^ > 2σ(*F*^2^)] = 0.039	Hydrogen site location: inferred from neighbouring sites
*wR*(*F*^2^) = 0.109	H-atom parameters constrained
*S* = 1.07	*w* = 1/[σ^2^(*F*_o_^2^) + (0.0489*P*)^2^ + 0.3284*P*] where *P* = (*F*_o_^2^ + 2*F*_c_^2^)/3
1145 reflections	(Δ/σ)_max_ = 0.001
87 parameters	Δρ_max_ = 0.34 e Å^−^^3^
0 restraints	Δρ_min_ = −0.57 e Å^−^^3^

### Special details

**Table:**

Geometry. All e.s.d.'s (except the e.s.d. in the dihedral angle between two l.s. planes) are estimated using the full covariance matrix. The cell e.s.d.'s are taken into account individually in the estimation of e.s.d.'s in distances, angles and torsion angles; correlations between e.s.d.'s in cell parameters are only used when they are defined by crystal symmetry. An approximate (isotropic) treatment of cell e.s.d.'s is used for estimating e.s.d.'s involving l.s. planes.
Refinement. Refinement of *F*^2^ against ALL reflections. The weighted *R*-factor *wR* and goodness of fit *S* are based on *F*^2^, conventional *R*-factors *R* are based on *F*, with *F* set to zero for negative *F*^2^. The threshold expression of *F*^2^ > σ(*F*^2^) is used only for calculating *R*-factors(gt) *etc*. and is not relevant to the choice of reflections for refinement. *R*-factors based on *F*^2^ are statistically about twice as large as those based on *F*, and *R*- factors based on ALL data will be even larger.

### Fractional atomic coordinates and isotropic or equivalent isotropic displacement parameters (Å^2^)

**Table:**

	*x*	*y*	*z*	*U*_iso_*/*U*_eq_	
Mn1	0.7500	0.7500	0.63202 (3)	0.0256 (2)	
N1	0.5501 (2)	0.81048 (17)	0.52125 (14)	0.0282 (5)	
N2	0.3599 (3)	0.87066 (19)	0.40742 (14)	0.0342 (5)	
H2	0.3136	0.8835	0.3500	0.041*	
C1	0.4022 (3)	0.87462 (18)	0.67505 (16)	0.0238 (5)	
C2	0.4063 (3)	0.86601 (18)	0.56583 (16)	0.0241 (5)	
C4	0.5155 (3)	0.8144 (2)	0.42597 (17)	0.0350 (6)	
H4	0.5895	0.7822	0.3775	0.042*	
C3	0.2882 (4)	0.9039 (2)	0.49557 (18)	0.0312 (5)	
H3	0.1804	0.9442	0.5057	0.037*	
O1	0.5374 (2)	0.83532 (14)	0.72111 (11)	0.0310 (4)	
O2	0.2642 (2)	0.91985 (15)	0.71532 (12)	0.0302 (4)	
O1W	0.5981 (2)	0.59207 (14)	0.66084 (14)	0.0392 (5)	
H1WA	0.6528	0.5466	0.7024	0.059*	
H1WB	0.5022	0.5856	0.6758	0.059*	

### Atomic displacement parameters (Å^2^)

**Table:**

	*U*^11^	*U*^22^	*U*^33^	*U*^12^	*U*^13^	*U*^23^
Mn1	0.0235 (4)	0.0344 (4)	0.0189 (3)	0.00646 (18)	0.000	0.000
N1	0.0272 (11)	0.0389 (12)	0.0184 (10)	0.0068 (8)	−0.0009 (8)	−0.0021 (8)
N2	0.0348 (13)	0.0490 (13)	0.0186 (10)	0.0037 (9)	−0.0074 (8)	0.0014 (8)
C1	0.0247 (12)	0.0233 (11)	0.0233 (12)	−0.0029 (9)	0.0020 (9)	−0.0011 (8)
C2	0.0243 (12)	0.0288 (12)	0.0192 (12)	0.0008 (9)	0.0002 (9)	−0.0007 (8)
C4	0.0359 (15)	0.0506 (16)	0.0186 (12)	0.0067 (11)	0.0000 (10)	−0.0031 (10)
C3	0.0289 (12)	0.0391 (13)	0.0256 (13)	0.0037 (10)	−0.0008 (10)	0.0009 (10)
O1	0.0287 (10)	0.0457 (10)	0.0186 (8)	0.0087 (7)	−0.0010 (7)	−0.0002 (7)
O2	0.0243 (10)	0.0432 (11)	0.0232 (10)	0.0047 (6)	0.0048 (6)	−0.0051 (7)
O1W	0.0279 (10)	0.0429 (11)	0.0467 (12)	0.0036 (7)	0.0039 (8)	0.0133 (8)

### Geometric parameters (Å, º)

**Table:**

Mn1—O1W	2.2037 (17)	N2—H2	0.8600
Mn1—O1W^i^	2.2038 (17)	C1—O1	1.257 (3)
Mn1—O1^i^	2.2079 (16)	C1—O2	1.264 (3)
Mn1—O1	2.2079 (16)	C1—C2	1.480 (3)
Mn1—N1	2.2097 (19)	C2—C3	1.359 (3)
Mn1—N1^i^	2.2097 (19)	C4—H4	0.9300
N1—C4	1.313 (3)	C3—H3	0.9300
N1—C2	1.377 (3)	O1W—H1WA	0.87
N2—C4	1.340 (3)	O1W—H1WB	0.73
N2—C3	1.359 (3)		
			
O1W—Mn1—O1W^i^	159.64 (10)	C4—N2—H2	126.1
O1W—Mn1—O1^i^	82.66 (6)	C3—N2—H2	126.1
O1W^i^—Mn1—O1^i^	86.27 (6)	O1—C1—O2	124.7 (2)
O1W—Mn1—O1	86.27 (6)	O1—C1—C2	116.93 (18)
O1W^i^—Mn1—O1	82.66 (6)	O2—C1—C2	118.34 (19)
O1^i^—Mn1—O1	113.90 (8)	C3—C2—N1	109.6 (2)
O1W—Mn1—N1	93.44 (7)	C3—C2—C1	131.4 (2)
O1W^i^—Mn1—N1	100.34 (7)	N1—C2—C1	118.98 (18)
O1^i^—Mn1—N1	168.96 (6)	N1—C4—N2	111.4 (2)
O1—Mn1—N1	75.97 (7)	N1—C4—H4	124.3
O1W—Mn1—N1^i^	100.34 (7)	N2—C4—H4	124.3
O1W^i^—Mn1—N1^i^	93.44 (7)	C2—C3—N2	105.9 (2)
O1^i^—Mn1—N1^i^	75.96 (7)	C2—C3—H3	127.1
O1—Mn1—N1^i^	168.97 (6)	N2—C3—H3	127.1
N1—Mn1—N1^i^	94.70 (10)	C1—O1—Mn1	116.82 (14)
C4—N1—C2	105.38 (19)	Mn1—O1W—H1WA	113.7
C4—N1—Mn1	143.43 (17)	Mn1—O1W—H1WB	128.2
C2—N1—Mn1	111.19 (14)	H1WA—O1W—H1WB	101.3
C4—N2—C3	107.8 (2)		
			
O1W—Mn1—N1—C4	−95.6 (3)	O1—C1—C2—N1	3.7 (3)
O1W^i^—Mn1—N1—C4	99.5 (3)	O2—C1—C2—N1	−175.9 (2)
O1^i^—Mn1—N1—C4	−26.7 (5)	C2—N1—C4—N2	1.0 (3)
O1—Mn1—N1—C4	179.1 (3)	Mn1—N1—C4—N2	−178.4 (2)
N1^i^—Mn1—N1—C4	5.1 (3)	C3—N2—C4—N1	−0.8 (3)
O1W—Mn1—N1—C2	85.01 (16)	N1—C2—C3—N2	0.3 (3)
O1W^i^—Mn1—N1—C2	−79.94 (16)	C1—C2—C3—N2	−179.3 (2)
O1^i^—Mn1—N1—C2	153.9 (3)	C4—N2—C3—C2	0.3 (3)
O1—Mn1—N1—C2	−0.28 (15)	O2—C1—O1—Mn1	175.69 (16)
N1^i^—Mn1—N1—C2	−174.31 (19)	C2—C1—O1—Mn1	−3.9 (2)
C4—N1—C2—C3	−0.8 (3)	O1W—Mn1—O1—C1	−92.12 (16)
Mn1—N1—C2—C3	178.83 (16)	O1W^i^—Mn1—O1—C1	105.01 (16)
C4—N1—C2—C1	178.8 (2)	O1^i^—Mn1—O1—C1	−172.39 (18)
Mn1—N1—C2—C1	−1.5 (2)	N1—Mn1—O1—C1	2.38 (16)
O1—C1—C2—C3	−176.7 (2)	N1^i^—Mn1—O1—C1	35.2 (4)
O2—C1—C2—C3	3.7 (3)		

Symmetry code: (i) −*x*+3/2, −*y*+3/2, *z*.

### Hydrogen-bond geometry (Å, º)

**Table:**

*D*—H···*A*	*D*—H	H···*A*	*D*···*A*	*D*—H···*A*
N2—H2···O2^ii^	0.86	1.95	2.811 (3)	173
O1*W*—H1*WA*···O2^iii^	0.87	1.96	2.818 (2)	167
O1*W*—H1*WB*···O2^iv^	0.73	2.02	2.751 (2)	176

Symmetry codes: (ii) −*x*+1/2, *y*, *z*−1/2; (iii) −*x*+1, *y*−1/2, −*z*+3/2; (iv) −*x*+1/2, −*y*+3/2, *z*.
